# Similar biodiversity of ectomycorrhizal fungi in set-aside plantations and ancient old-growth broadleaved forests

**DOI:** 10.1016/j.biocon.2015.12.003

**Published:** 2016-02

**Authors:** Rebecca Spake, Sietse van der Linde, Adrian C. Newton, Laura M. Suz, Martin I. Bidartondo, C. Patrick Doncaster

**Affiliations:** a Centre for Biological Sciences, Institute for Life Sciences Building 85, University of Southampton, Southampton, SO17 1BJ, UK; b Department of Life Sciences, Imperial College London, London, SW7 2AZ, UK; c Comparative Plant and Fungal Biology, Jodrell Gate, Royal Botanic Gardens, Kew, Richmond, TW9 3DS, UK; d Centre for Ecology, Environment and Sustainability, Faculty of Science and Technology, Bournemouth University, Talbot Campus, Fern Barrow, Poole, Dorset BH12 5BB, UK

**Keywords:** Ectomycorrhizal fungi, Oak, Old-growth, Overmature, Planted forest, Set-aside, Species richness, Sporocarp

## Abstract

Setting aside overmature planted forests is currently seen as an option for preserving species associated with old-growth forests, such as those with dispersal limitation. Few data exist, however, on the utility of set-aside plantations for this purpose, or the value of this habitat type for biodiversity relative to old-growth semi-natural ecosystems. Here, we evaluate the contribution of forest type relative to habitat characteristics in determining species richness and composition in seven forest blocks, each containing an ancient old-growth stand (> 1000 yrs) paired with a set-aside even-aged planted stand (ca. 180 yrs). We investigated the functionally important yet relatively neglected ectomycorrhizal fungi (EMF), a group for which the importance of forest age has not been assessed in broadleaved forests. We found that forest type was not an important determinant of EMF species richness or composition, demonstrating that set-aside can be an effective option for conserving ancient EMF communities. Species richness of above-ground EMF fruiting bodies was principally related to the basal area of the stand (a correlate of canopy cover) and tree species diversity, whilst richness of below-ground ectomycorrhizae was driven only by tree diversity. Our results suggest that overmature planted forest stands, particularly those that are mixed-woods with high basal area, are an effective means to connect and expand ecological networks of ancient old-growth forests in historically deforested and fragmented landscapes for ectomycorrhizal fungi.

## Highlights

• Ectomycorrhizal fungi richness was similar in ancient and overmature planted forest.
• Community composition was also similar across the forest types.
• Fungal fruit body richness was positively related to basal area and tree diversity.
• Below-ground ectomycorrhizae richness was positively related to tree diversity.

## 1 Introduction

High structural diversity and long continuity of old-growth forests make them irreplaceable biodiversity resources (Gibson et al., 2011, Spake et al., 2015). Planted or regenerated forests of relatively young and even age now constitute 73% of total forest cover in Europe (San-Miguel-Ayanz et al., 2011). Throughout Europe, timber-orientated forest management has shortened forest stand development to just 10–40% of the potential lifespan of dominant tree species (Bauhus et al., 2009, Barbati et al., 2012). These forests consequently lack the continuity and many of the structural attributes typical of old-growth forests, including high variation in tree size, the presence of large dying trees and irregular gap size and distribution (Bauhus et al., 2009).

Concern about the global decline of old-growth forest and the associated loss of biodiversity has motivated initiatives to increase the area of protected old-growth (CBD, 2011). Although strict protection of natural forests will likely remain a conservation priority throughout the world, management options increasingly recognise the additional potential for other types of forests to support biodiversity (Gibson et al., 2011, Putz et al., 2012). Presently, there is much interest in the setting aside of overmature planted stands as a means of preserving species associated with old-growth forests (Humphrey, 2005, Lassauce et al., 2013). Set-aside is a topical forest management strategy in Europe, where countries generally have a greater area of even-aged planted forests approaching biological maturity than old-growth forest (Barbati et al., 2014). The ageing of even-aged forest stands is a continuous process in which small-scale disturbances shape forest structural diversification (Barbati et al., 2012), with consequences for biodiversity conservation, carbon storage and the ways energy, gases and nutrients are cycled through the forest (Parker et al., 2004).

Species associated with old-growth forest are predicted to accumulate over time in set-aside in response to the increasing structural diversification as a forest ages. The temporal continuity and ever-enriching diversity of resources particularly favours colonisation by the dispersal-limited species characteristic of old-growth forest (Norden and Appelqvist, 2001). Indeed, several studies of dispersal-limited groups have demonstrated that ecological communities from older forests are generally, but not invariably, richer than those of more recent forests. For example, Peterken (1974) and Rose (1974) showed that the occurrences of certain vascular plants and epiphytic lichens could distinguish ancient semi-natural forests (old-growth forest that has been continuously wooded since 1600 a.d.) from more recent, secondary forest stands. Whilst differences in species composition between ancient and more recent forests can be attributed to differences in stand structure and microhabitat composition, many studies emphasise the importance of time per se; evidenced by certain species being absent from young forests despite the presence of suitable habitats (Norden and Appelqvist, 2001). For example, many cyanobacterial macrolichens are rare in recent stands even if seemingly suitable substrate is available (Kuusinen, 1996). It is therefore thought to be the longer residence time that is important in allowing dispersal limited species to reach and establish in a habitat (Norden and Appelqvist, 2001).

Despite the perceived importance of overmature planted forest in supporting old-growth forest communities, empirical data are lacking on their relative biodiversity values for many taxonomic groups (Sverdrup-Thygeson et al., 2015). A recent meta-analysis synthesising species richness relationships with stand age in temperate and boreal forests demonstrated functional group-specific responses to stand age, owing to specific dependencies on resources or environmental conditions that become available at different times during succession (Spake et al., 2015). This synthesis showed that species richness in planted and regenerating forests can eventually achieve equivalence to old-growth forests for some functional groups, including epiphytic lichens and fungi. Compositional differences have yet to be investigated, however, and the potential for planted forests to support old-growth forest communities remains poorly understood. Here we investigate empirically the value of planted, economically overmature forests to ectomycorrhizal fungal communities, relative to ancient old-growth forest in the New Forest National Park, UK.

Ectomycorrhizal fungi (EMF) comprise a functional group that forms mutualistic associations with most economically and ecologically important temperate tree species (Smith and Read, 2008). Approximately 90% of species rely on mycelial networks intimately connected with their roots, ectomycorrhizae, for the uptake of water and N, P, and other minerals from soil (Heilmann-Clausen et al., 2014). EMF form a highly diverse biota in boreal and temperate forests, which is sensitive to natural and anthropogenic disturbances, such as nitrogen deposition (Barker et al., 2013, Suz et al., 2014). High EMF diversity is important both to the vigour of individual hosts and to the functioning of the forest ecosystem. At the individual level, the diverse capacities amongst EMF species for mobilising nutrients from soil mineral and organic matter (Nygren et al., 2007) insure a host tree against environmental stresses (Courty et al., 2010). At the ecosystem level, EMF are not only important for nutrient cycling, but high EMF diversity can facilitate resistance to disease and drought (Kernaghan, 2005), and contribute to net primary productivity, mineral weathering and soil carbon storage (Smith and Read, 2008).

Whilst the relative biodiversity value of ancient old-growth and more recent secondary forest has been established for groups including vascular plants and epiphytic lichens (Rose, 1974, Peterken, 1974), we still lack sufficient empirical assessments of the relative biodiversity value of planted and old-growth forest for EMF. To date, studies have reported only from coniferous forest in North America (e.g. Kranabetter et al., 2005, Twieg et al., 2007), where they show that EMF exhibit much lower species richness in young secondary forests. Meta-analysis of these studies shows an average time of 90 years to recover EMF richness to old-growth values (between 45 years to unrecoverable at 95% prediction limits: Spake et al., 2015). No previous study has examined EMF-stand age relationships in broadleaved forests with a statistically robust study design. Dispersal limitation is important in structuring EMF communities, despite the fact that fungal fruit bodies produce large numbers of spores with high potential for long distance travel (Peay et al., 2010). Indeed, red-lists and field observations suggest that numerous species are likely to be confined to old-growth forests due to infrequent dispersal (Dickson and Leonard, 1996, Senn-Irlet et al., 2007).

In historically deforested regions such as the UK, where the proportion of forest has declined to just 12% of total land area, ~ 80% of this forested area is planted and just 5% is classified as ancient (UK National Ecosystem Assessment, 2011), the setting-aside of planted forests presents a particularly pertinent opportunity for increasing the area of forest that can support the biodiversity and functions that characterise old-growth forests (Bauhus et al., 2009). The aims of this study were to (1) compare EMF biodiversity and species associations amongst overmature planted and ancient old-growth forest stands; and (2) determine what stand characteristics explain the differences in species richness and composition in order to identify opportunities for enhancing EMF diversity in overmature planted stands.

## 2 Methods

### 2.1 Study area

The study area was located in the New Forest National Park in southern England, UK (Figure A1.1). The New Forest lies within the Warm Dry climatic zone (Pyatt et al., 2001), with mild winters and warm summers. Temperatures range from 2.3 °C (mean February minima) to 20.8 °C (mean July maxima). Annual precipitation is 760 mm. Soils are mainly brown earths above Barton clays and Chama sands overlying Tertiary gravels. The old-growth forests in this study are ancient sensu Peterken (1977), in that they have originated before a threshold date of 1600 A.D.; a time before which secondary forests were rarely established through planting. In fact, the New Forest's ancient forests include remnants of post-glacial forest that have never been completely cleared (Tubbs, 2001), and the ancient stands under study all had stand continuity of > 1000 years. They are also deemed semi-natural, in that they have been selectively felled for timber in the past, but allowed to regenerate naturally, without any major tree removal since the mid-20th century. The canopies of the ancient and planted stands under study are dominated by English oak (*Quercus robur*), sessile oak (*Quercus petraea*) and beech (*Fagus sylvatica*) with the understorey consisting largely of holly (*Ilex aquifolium*) (Tubbs, 2001).

The plantations under study were on average 180 years old. Plantation ages were considered equal to the number of years since planting (Table A1.1; Appendix A2). Plantations were established following clear-cutting of partially forested (deer parks) to completely forested grounds. All sites likely underwent the same preparation procedure following clear-cutting, involving the upturning of soil and trench formation for water drainage (see Appendix A2 for a detailed description). The management histories of the ancient old-growth stands under study were elucidated using historic maps, Forestry Commission management plans, pollen records and consultation with local experts. The stand ages refer to the oldest record available indicating that the site was forested (Table A1.1), and since then have not experienced major tree removal.

### 2.2 Study design

In order to compare EMF communities in overmature planted set-asides and ancient old-growth forest communities, locations were selected that paired forest types in a randomised block design (Hurlbert, 1984). Seven forest locations containing both a planted stand and an ancient stand were identified. In order to minimise extraneous variation, paired stands were matched for canopy species (oak dominated), elevation and underlying geology, and separated by < 1 km. Within each stand at each location, five 10 m × 10 m permanent assessment plots were selected inside a 2 ha area using stratified random coordinates. Stratification met selection criteria of oak dominance, homogeneous tree and vegetation cover, relatively flat topography and absence of atypical or exotic tree species.

### 2.3 Sampling of EMF communities

We used two methods to obtain representative samples of EMF communities in plots: surveys of above-ground sporocarps (‘mushrooms’), and soil cores for analysis of below-ground EMF on root tips (Taylor, 2002; henceforth ‘ectomycorrhizae’).

#### 2.3.1 Above-ground sampling of sporocarps

The incidence of all visible above-ground EMF macrofungal sporocarps was recorded across all assessment plots. We define macrofungi as species with fruiting bodies large enough to be collected in the field without the aid of a hand lens, including the majority of basidiomycetes and many ascomycetes (Balmford et al., 2000). Plots were surveyed during autumn 2012, 2013 and 2014 (September–November) to coincide with the main time of sporocarp production. Three visits were made to each plot at approximately monthly intervals over this period. Species identifications were made using a compound microscope and standard texts (Phillips 2006; Moser 1983), and more specialised texts for *Lactarius* (Heilmann-Clausen et al., 1998), *Inocybe* (Outen and Cullington, 2009), *Cortinarius* (Knudsen and Vesterholdt, 2012), *Russula* (Kibby, 2012c), *Boletus* (Kibby, 2012a), and *Amanita* (Kibby, 2012b). To obtain a measure of species richness, sporocarp incidence data were pooled to the plot level across all plot visits.

#### 2.3.2 Below-ground sampling of ectomycorrhizae

Below-ground sampling took place between February and July 2014, in locations 1–4 only (Table A1.1). These were a random selection of the available seven locations. Each plot was divided into a 10 × 10 grid, and every intersection given a coordinate. Sixteen soil cores (2 cm diameter, 30 cm length) were taken at randomly generated coordinates. Samples were stored at 4 °C until root tip analysis could be undertaken, always within 5 days. Cores were soaked in tap water for 15 min and washed through a 500 μm sieve. After manual removal of coarse woody debris and senescent roots, live roots were extracted from the core for five minutes under a dissecting microscope. To minimise observer bias, three of the largest roots were selected, and one living root tip was randomly selected from each of these following Cox et al. (2010) and Suz et al. (2014). This sampling intensity was justified from the results of preliminary sampling in January 2014, which recorded > 60% of the Chao2 estimate of EMF species (Figure A3).

#### 2.3.3 Molecular identification of ectomycorrhizae on root tips

Identification of ectomycorrhizae followed the methodology of Suz et al. (2014). Fungal DNA was extracted from the selected EMF tips with the Extract-N-Amp™ Plant PCR kit (Sigma-Aldrich, St. Louis, USA) with some modifications of the protocol: EMF tips were incubated in 8-μL of extraction solution and diluted in 8-μL of dilution solution. The internal transcribed spacer (ITS) region of the rDNA was amplified by polymerase chain reaction (PCR) on 0.5 μL of the freshly extracted DNA, mixed with – 4 μL of the Extract-N-Amp PCR ReadyMix™ (Sigma-Aldrich, St. Louis, USA), 0.4 μL of each primer at 10 μM and 3.4 μL of distilled water. All PCR reactions were performed using the primer pair ITS1-F (Gardes and Bruns, 1993) and ITS4 (White et al., 1990).

Fungal ITS sequences were analysed and edited with Geneious version R7, Biomatters, available from http://www.geneious.com/. Edited sequences were identified using the BLAST algorithm in GenBank and the UNITE database (http://unite.ut.ee/). The best BLAST identification was reported for each fungal taxon. The UNITE species name was validated only when (i) the similarity between the submitted sequence and the sequence in the database exceeded 97%, and (ii) the UNITE identification was plausible taking into account ecological considerations and known geographical distributions of related species (Richard et al., 2011). Sequences with < 97% of similarity with the nearest blast, or for which the UNITE species name was considered too uncertain, were ascribed to an undetermined operational taxonomic unit (OTU).

### 2.4 Quantification of environmental variables

Soil was sampled from all plots during a consistently dry period with minimal spatio-temporal variation in rainfall during May 2014. Five random soil cores (2 cm diameter, 30 cm length) were taken from each 10 m × 10 m fungal assessment plot and were pooled to the stand level. Samples were sent to the Forest Research Soil Analysis Service (Surrey, UK), for analysis. The following soil variables were measured: (i) pH; (ii) moisture content (loss in fresh mass after 2 day at 105 °C); (iii) organic matter (loss on ignition after 1 day at 450 °C); (iv) water-soluble anions, NO_3_–N, NH_4_–N, PO_4_–; (v) exchangeable cations, K +, Mg 2+, Ca 2+; (vi) total N; and (vii) total C. See Humphrey et al. (2003) for further details of soil analytical procedures. All vascular plants rooted in the plots below 1 m in height were sampled to obtain a measure of understorey richness (Gilliam, 2007). For stand structural assessments, the 10 m × 10 m plots were nested within 30 m × 30 m plots. Each 30 m × 30 m plot was assessed for stand basal area, tree species diversity (Shannon–Weiner, using basal area as a measure of species' relative abundances) and canopy closure. Assessments of diameter at breast height were inclusive of all trees > 5 cm diameter and followed standard protocols (Newton, 2008). Canopy closure, the proportion of the sky hemisphere obscured by vegetation when viewed from a single point (Jennings et al., 1999), was estimated by taking the average of five measurements using a spherical densiometer at each plot corner and centre. Details of stand environmental variables are given in Table A2.1.

### 2.5 Statistical analysis

#### 2.5.1 Variation of species richness with forest type and other environmental variables

All analyses were computed in R 3.00 software (R Core Team, 2013). Prior to analysis, environmental variables were centred and scaled to improve the interpretability of regression coefficients, following Schielzeth (2010). Basal area was square rooted. Principal component analysis (PCA) was used to produce orthogonal axes (Soil1, Soil2, Soil3), representing > 70% of variation in the soil chemistry data for both sporocarp and ectomycorrhizae datasets. Because not all EMF root tips were sequenced successfully, below-ground sampling intensity was unequal across plots. We therefore estimated individual-based sporocarp species richness at plot-level with Chao1 (Chao, 1984), using the vegan package (Oksanen et al., 2013).

The simultaneous effects of environmental variables on EMF richness were quantified using mixed effects models. Two response variables were investigated: sporocarp and ectomycorrhizae richness, the former using a generalized mixed model with Poisson error distribution and logarithmic link function, and the latter using a linear mixed model on log-transformed Chao1 estimated richness. Explanatory variables included: forest type (planted or ancient old-growth), tree species richness, understorey species richness, tree basal area, Soil1, Soil2 and Soil3. Canopy closure was not included due to co-linearity with the more precisely measured basal area (Pearson's *r* = 0.80, *P* < 0.001).

Location was included as a random factor crossed with forest type, reflecting the designed pairing of the two types in each location. All possible additive models were constructed by maximum likelihood methods using packages lme4 (Bates et al., 2014), and MuMIn (Barton, 2013). Akaike's Information Criterion (AIC) with small sample correction bias (AICc) was used to identify the best model, and all plausible models with ∆ AICc < 7 (Burnham and Anderson, 2004, Aho et al., 2014). Random intercepts were featured in the mixed models only, as allowing for slopes caused a large positive ∆ AICc. Goodness of model fits of the minimum adequate model and other plausible models was estimated by calculating the marginal *R*^2^, following Nakagawa and Schielzeth (2013). The relative importance values of the explanatory variables were calculated by summing up the Akaike weights of all plausible models (with ∆ AICc < 7) that included the variable in question (Burnham and Anderson, 2004).

#### 2.5.2 Community composition in planted and ancient old-growth stands

All analyses of community composition used the ‘vegan’ R package (Oksanen et al., 2013). Tests of compositional differences between planted and ancient forest types used a non-parametric multi-response permutation procedure (MRPP) (Zimmerman et al., 1985), based on the Sørensen (Bray–Curtis) distance measure (McCune and Grace, 2002, Promis et al., 2012). Its statistic *A* describes within-group homogeneity relative to random expectation. *A* = 1 signifies that all items are identical within groups; *A* = 0 signifies that heterogeneity within groups equals chance expectation; *A* < 0 signifies less agreement within groups than chance expectation (Promis et al., 2012).

Differences in EMF composition across forest types were visualised with nonmetric multidimensional scaling (NMDS). NMDS reflects similarities (or dissimilarities) between assemblages. The Sørensen distance measure was used for species presence–absence data. Two-dimensional solutions were produced with 999 iterations, as the reduction in stress was small beyond the second axis.

Species fidelities to each forest type were identified by indicator species analysis (ISA; Dufrêne and Legendre, 1997) extended by De Caceres et al. (2010), using package IndicSpecies (De Cáceres and Legendre, 2009). Indicator values determine how strongly each species associates to a forest type, based on two probabilities: (i) the probability that a surveyed plot belongs to the target forest type (specificity), and (ii) the probability of finding the species in plots belonging to the forest type (sensitivity). Indicator values range from 0 (no indication) to 1 (maximum indication). Statistical significances of indicator values were tested using a randomisation procedure based on 999 permutations.

We estimated the Chao similarity index to quantify the compositional similarity between EMF communities sampled as sporocarps and ectomycorrhizae following Chao et al. (2005). We used the proportion of sampled plots that species occurred in as a measure of relative abundance. This method calculates a Jaccard- or Sørensen-type dissimilarity index that accounts for the effect of unseen shared species, based on replicated incidence or abundance sample data, respectively. Values range between 0 (no similarity) and 1 (complete similarity).

## 3 Results

A total of 225 EMF taxa were identified across all plots, with 122 species identified in the sporocarp survey and 136 OTUs characterised using the molecular analysis (Appendix A4). The same four species were most frequently observed in the sporocarp and ectomycorrhizae surveys, in terms of the percentage of plots containing them: *Lactarius tabidus* (79% sporocarp, 37% ectomycorrhizae), *Lactarius quietus* (74%, 48%), *Laccaria amethystina* (73%, 29%) and *Russula ochroleuca* (53%, 31%). Visual examination of species' ranked abundance distributions suggests that the EMF communities from both forest types followed a Zipf–Mandelbrot distribution, indicating that communities have a few species that are very abundant, and a long tail of rarer species (Figure A4.1). The ancient old-growth stand at location 2 and the planted stand at location 7 were disregarded from the analysis, after the PCA analysis of soil chemistry revealed unrepresentative soil conditions in extreme outliers (Figure A5.1).

### 3.1 Environmental drivers of EMF richness

**Fig. 1:**
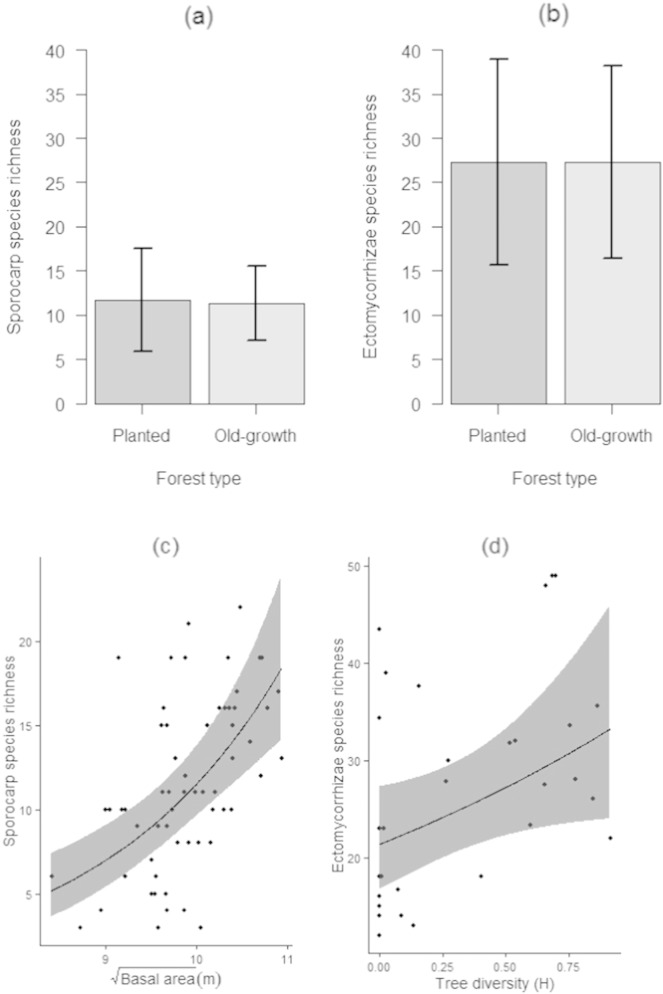
The influence of forest type, (a) and (b), and the most important environmental variables on EMF richness (c) and (d). Neither sporocarp (a) nor ectomycorrhizae (b) richness varied detectably by forest type (bars show mean + SD). (c) Influence of square-rooted basal area on sporocarp richness; (d) influence of tree diversity (Shannon–Wiener H) on richness of ectomycorrhizae. Regression coefficients are for minimum adequate models based on AICc, with 95% prediction interval (grey shading) based on uncertainty in the fixed effects.

Forest type was not found to be an important determinant of either sporocarps or ectomycorrhizae richness, as shown by its low relative importance values, summed over plausible models with ∆ AICc < 7 (Table 1, Table 2, Fig. 1a & b). The most important variables explaining EMF richness were basal area and tree diversity (Table 1, Table 2). For sporocarps, basal area was the most important predictor of species richness (Table 1a), with a strong positive effect (Fig. 1c) and a relative importance value of 1.00 (Table 2). For ectomycorrhizae, tree diversity was a major positive driver of EMF richness (Table 1b, Fig. 1d), which had a relative importance value of 0.60 (Table 2) and was the only variable included in the minimum adequate model (Table 1b) explaining below-ground richness. Marginal *R*^2^ values were higher for models explaining sporocarp richness (0.30 for the best model; Table 1a) than below-ground richness (0.15 for the minimum adequate model; Table 1b).

Sporocarp richness was also positively driven by tree diversity and understorey richness (importance values of 0.54 and 0.40; Table 2), and the richness of ectomycorrhizae was also influenced by Soil2 and Soil3, though moderately so (relative importance values 0.47 and 0.39; Table 2). Soil2, the second PCA axis of the soil chemistry data, indicated a gradient of increasing ratio of carbon to nitrogen, and decreasing ammonium, sulphate and soil pH. See Appendix A6 for a more detailed description of the soil PCA axes.

### 3.2 EMF community composition

**Fig. 2:**
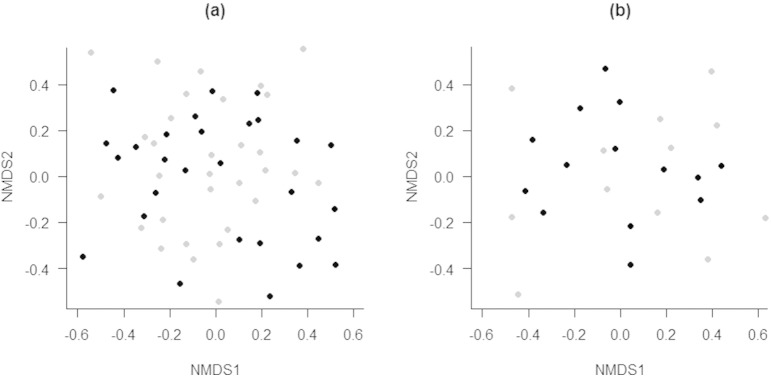
NMDS ordination on (a) sporocarp and (b) ectomycorrhizae communities, using Sørensen distance of ancient (black) and overmature planted (grey) forest plots based on plot-level EMF presence-absence data.

MRPP analysis revealed no differences in EMF species composition between overmature planted and ancient old-growth forest plots, for communities of either sporocarp or ectomycorrhizae (*A* < 0.01, *P* = 0.41 and *A* = 0.00, *P* = 0.73, respectively). The NMDS ordination confirmed these results, finding no separation in species composition according to forest type (Fig. 2; *R*^*2*^ = 0.02; *P* = 0.28 and *R*^*2*^ = 0.00; *P* = 0.91 for sporocarp and ectomycorrhizae, respectively). A two-dimensional solution described 63% and 66% of the variance in the species composition for sporocarp and ectomycorrhizae datasets, respectively (Fig. 2).

Indicator species analysis detected associations of a single species with each forest type from the sporocarp surveys, and of a single species with ancient old-growth forest from the ectomycorrhizae samples (Table 3). Indicator values were only moderate, however (Table 3; De Cáceres and Legendre, 2009). Two of the species recorded are on the Red List for British fungi (Evans et al., 2006). Both species, *Cortinarius orellanus* (Vulnerable) and *Cortinarius violaceus* (Near Threatened), were found as sporocarps in ancient old-growth stands at locations 1 and 2, respectively. Due to their incidences within single plots, they had low sensitivities to forest type, and so did not have significant indicator values.

Compositional similarity between communities sampled as sporocarps and ectomycorrhizae was only moderate with Chao similarity indices of 0.41 for ancient and 0.37 for planted forest (Chao et al., 2005).

## 4 Discussion

### 4.1 The effectiveness of set-aside overmature planted forest at conserving ancient forest ectomycorrhizal communities

This investigation found similar richness and composition of EMF in set-aside plantation and neighbouring ancient stands. It has been suggested that increasing the proportion of overmature planted forests could offer alternative habitats for species typical of old-growth forests, but this possibility lacks empirical evaluation (Martikainen et al., 2000, Sverdrup-Thygeson et al., 2015). Many EMF species require long periods of stand continuity for colonisation events to recover richness to old-growth levels, consistent with strong dispersal limitation. Despite the fact that fungal fruit bodies produce large numbers of spores with high potential for long-distance travel, relatively recent evidence suggests that dispersal limitation is significant in EMF assemblages (Peay et al., 2010). A meta-analysis of EMF recovery in planted and secondary forests showed that recovery of species richness is possible after ~ 90 years in temperate and boreal regions (Spake et al., 2015). This estimate, however, is based exclusively on data from coniferous forest, and from secondary forests less than a century old. Our empirical study has shown that set-aside overmature broadleaved plantations ~ 180 years old can attain the species richness of old-growth semi-natural forests > 1000 years old.

The EMF communities in the overmature plantations were indistinguishable from those of ancient old-growth forests (Fig. 2). Our findings concord with studies of regeneration following harvest of mixed coniferous forests in the USA (Twieg et al., 2007) and tropical forest in China (Gao et al., 2015). Both of these studies found significant changes in the compositions of EMF communities between young and intermediate-aged or old forest stands, but no significant differences between intermediate-aged and old forests.

EMF communities observed within samples of sporocarps and ectomycorrhizae had only moderate overlap in species composition, in contrast to the complete overlap between forest types. Other studies using both methods often report a poor correspondence between them in terms of species richness and community composition (Dahlberg et al., 1997, Horton and Bruns, 2001). Such differences have been attributed to differences in modes of reproduction, but also differences in methodological caveats (discussed below) and sampling effort between the techniques; sampling of ectomycorrhizae typically spans a much a shorter time period than sporocarp surveys (five months vs. three years in our study). Indeed, temporal partitioning amongst EMF species has been observed (Koide et al., 2007), which may cause many species to be missed by the short and infrequent sampling of ectomycorrhizae on root tips (Toth and Barta, 2010).

Different variables were important in predicting sporocarp and ectomycorrhizae species richness patterns, with sporocarp richness driven by basal area as a proxy for canopy closure and tree diversity, and the richness of ectomycorrhizae driven by tree diversity only (Fig. 1). It is possible that these differences may reflect differences in the communities sampled by above-ground sporocarp and below-ground ectomycorrhizae sampling methods. Up to 80% of EMF biomass in forest soils is in the form of external mycelia (Wallander et al., 2001). Patterns of resource allocation by a fungus to the production of sporocarps vs. ectomycorrhizal root tip formation vary amongst species (Gardes and Bruns, 1996). It is therefore likely that the sampled above-ground richness is more responsive than below-ground richness to changes in carbon allocation, which increases with canopy closure: at canopy closure, tree growth rates are rapid and leaf area maximal, with correspondingly high potential for carbon allocation to roots and ectomycorrhizal partners (Twieg et al., 2007). It is not surprising that EMF richness increased with tree diversity; different tree hosts provide unique habitats for host-specific taxa (Tedersoo et al., 2012).

### 4.2 Methodological caveats

We sampled both sporocarps and ectomycorrhizae in our attempt to acquire an accurate unbiased representation of the EMF communities within our plots. Each technique has limitations. Sporocarp production is sensitive to a host of environmental factors, and consequently sporadic annual fruiting patterns necessitate long term monitoring (Dahlberg et al., 1997). In mesic temperate climates, 3–8 years is considered the minimum sampling period necessary to obtain a reasonable representation of fungal community structure (Vogt et al., 1992, Gardes and Bruns, 1996). We sampled only macrofungal sporocarps, those that are large enough to be collected in the field without the aid of a hand lens (Balmford et al., 2000); but the sporocarps of many species are inconspicuous (e.g. corticioid species) or hypogeous (e.g. truffles; Peter et al., 2001,, leading to underestimates of diversity if sporocarp surveys are used alone. The sampling of ectomycorrhizae in our study complemented the sporocarp surveys and enabled the discovery of species that are inconspicuous above-ground e.g. *Cenococcum geophilum*. However, the molecular sequencing of ectomycorrhizae is expensive and time-consuming resulting in relatively small volumes of soil being screened.

Sporocarp surveys are essential for detecting those rare species that form conspicuous sporocarps (Smith et al., 2002). It is thought that there is high variation in fruiting activity amongst species, ranging from annual fruiting, where species rely heavily on spores for propagation, e.g. *Lactarius* and *Russula*; or sporadic fruiting, in which species do not produce sporocarps every year (O'Hanlon, 2012). Smith et al. (2002) have suggested that older forest stands comprise rare species that fruit infrequently, due to decreasing net primary production (NPP) and corresponding below-ground carbon allocation with stand age. They compared sporocarp richness and composition in young, rotation-age, and old-growth stands of Douglas-fir (*Pseudotsuga menziesii*) in Canada, and found old-growth stands contained many species of fungi that infrequently produce sporocarps. With sporocarp surveys disadvantaged by the inherent sporadicity of EMF fruiting and ectomycorrhizae surveys limited by the small volume of soil sampled, either sampling method may have omitted the inclusion of rarer EMF species that fruit infrequently. Indeed, two species of conservation concern were recorded only once, both in ancient old-growth stands. It is possible that further sampling may reveal more rare species, particularly within ancient stands: Smith et al. (2002) sampled over 4 years, whilst we present 3 years of sampling.

A study of EMF communities in forests across the UK in the 1990s (Humphrey et al., 2000) surveyed 100 m × 100 m plots each within eight stands of ‘mature’ to ‘overmature’ oak forest (~ 200 years) for sporocarps over 3 years, and found a mean of ~ 14 species per stand. Comparing this value with the mean of ~ 30 species per stand from sporocarp surveys in this study (after pooling the five, smaller 10 m × 10 m plots to the stand level), we are confident that our sampling was adequate in obtaining a representative sample of EMF richness. Species accumulation curves using both methods support this assertion (Figure A3.2). Furthermore, the combination of two sampling techniques, used simultaneously across replicate sites of interest, increases the validity of the comparisons we have drawn between communities from ancient and set-aside planted stands.

It is possible that the ancient old-growth forests of the New Forest National Park comprise a depauperate subset of the EMF community that was once supported in the past. Indeed, potential threats to EMF including nitrogen deposition, selective tree felling and soil compaction by high herbivore densities (Newton, 2010), might have diminished EMF diversity in the park. If this were the case, the generalization of our results to other areas where EMF diversity has not been diminished may be inappropriate. Nevertheless, the New Forest has been designated as an Important Fungus Area by national assessment, due to its high diversity (~ 2600 species of fungi across all functional groups and habitat types have been recorded across the New Forest; Dickson and Leonard, 1996), the persistence of populations of conservation concern and the presence of habitats of known mycological importance (Evans et al., 2001, Newton, 2010).

### 4.3 Conservation and management implications

The setting aside of overmature planted forest is an effective means of conserving EMF communities associated with ancient old-growth forests, given temporal continuity of the order of a century. Whilst this is the first study to investigate the importance of ecological continuity for EMF in overmature broadleaved plantations, similar studies have been done for other taxonomic groups; Carpenter et al. (2012) sampled soil, litter and ground invertebrates from ancient and overmature planted broadleaved forest stands across the New Forest and found that species richness and community composition did not differ amongst the forest types. This holds out particular promise for historically deforested regions such as the UK, where little ancient forest remains and much planted forest exceeds a century in age. In our study, the paired ancient and set-aside planted stands were separated by less than 1 km. Given that many EMF are dispersal limited, it is possible that the ability of overmature plantations to function as reservoirs for old-growth EMF communities may depend on the degree of connectivity with old-growth propagule sources. Indeed, Humphrey et al. (2004) observed a negative relationship between EMF species richness of conifer plantations with distance from the nearest ancient woodland across the UK. This finding has relevance to the development of ecological networks, a major policy driver in many countries that aims to mitigate against biodiversity loss in highly fragmented landscapes (Jongman, 2004, Opdam et al., 2006, Lawton et al., 2010). Ecological networks represent a suite of core areas of habitat connected by buffer zones, corridors and smaller stepping stone patches that allow movement of species or their propagules (Lawton et al., 2010, Humphrey et al., 2015). Our study suggests that set-aside, overmature planted stands can function as effective stepping stones in connecting ancient forest stands. The protection of overmature planted forest stands located near or adjacent to ancient semi-natural forest should therefore represent a conservation priority.

Our study has identified influences of habitat variables that suggest opportunities for enhancing EMF diversity in planted forest. EMF distribution and composition have been shown to be influenced by the relative proportions of host tree species, indicating a degree of host preference or specificity for some EMF species or genera (Newton and Haigh, 1998). In this study, tree diversity was important in driving sporocarp and ectomycorrhizae richness (Fig. 1). Correlations between host richness and EMF richness are a common finding in empirical research comparing monocultures with mixed woods (Cavard et al., 2011), probably due to a higher host richness providing more unique habitats for host-specific taxa (Tedersoo et al., 2012), or more facilitation of EMF taxa that associate with multiple hosts (Cavard et al., 2011). Our findings support sustainable forest management strategies that promote mixed-wood management, which will likely enhance EMF richness. Understorey richness was also important in driving up sporocarp richness, suggesting that restoration of plant communities, a major goal of forest restoration efforts, may simultaneously enhance EMF sporocarp richness.

Basal area correlated positively with sporocarp richness (Fig. 1). Although we cannot distinguish between the effects of basal area and canopy closure (with which it strongly correlates), the positive response is likely due to the combined effects of increasing density of roots (and therefore a function of the species-area relationship), and the associated increased carbon availability for EMF partners with increasing canopy closure (Twieg et al., 2007). Furthermore, EMF richness patterns revealed by sporocarp surveys also shed light on the influence of environmental variation on sporocarp production, on which canopy closure has been shown to be a key driver, as it affects precipitation interception (Santos-Silva et al., 2011). Increases in tree stress and mortality and associated declines in basal area have been observed in both managed and unmanaged forest stands across the world (Allen et al., 2010, Mcintyre et al., 2015). These are driven by direct impacts of climate change on drought frequency and severity (Gonzalez et al., 2010), and on the dynamics of forest insects and pathogens (Allen et al., 2010), and by inhibited regeneration due to recreation and over-grazing (Mountford and Peterken 2003). Our results suggest that EMF richness will likely benefit from conservation measures designed to sustain basal area within set-asides and ancient stands.

## Role of funding sources

The Biology and Biotechnology Research Council (grant no. BB/H531935/1) and British Mycological Society (‘Eunice Jones Bequest Fund’ and ‘Small grant’) provided funding to RS, NERC (NE/K006339/1) provided support to MIB and SVDL, and an EU Marie Curie IEF fellowship (FP7-PEOPLE-2009-IEF - 253036 - MYCOIND) provided support to LMS.

## Figures and Tables

**Table 1:** Fixed variables included in mixed models explaining variation in sporocarp and ectomycorrhizae species richness of overmature planted and ancient old-growth forest stands (‘+’ indicates the variable's inclusion). Only models with ∆ AICc < 2 are shown. See methods for model details.

Variables included in model								df	∆ AICc	Marginal *R*^2^
	Forest type	Basal area	Tree div	Understorey richness	Soil1	Soil2	Soil3			
*(a) Sporocarp*										
1		+	+					4	0.00	0.30
2		+	+	+				6	0.26	0.32
3		+						4	0.83	0.24
4		+			+			5	1.19	0.29
5		+		+				5	1.41	0.26
6		+	+		+			6	1.67	0.32
7	+	+						5	1.72	0.30
8		+	+			+		6	1.73	0.29
9	+	+	+					6	1.79	0.29

*(b) Ectomycorrhizae*										
1			+					5	0.00	0.15
2			+			+		6	0.19	0.27
3						+	+	6	0.85	0.27
4			+			+	+	7	1.11	0.34
5								4	1.13	0.00
6			+				+	6	1.54	0.26
7	+		+					6	1.60	0.21

**Table 2:** Relative importance values for explanatory variables contained within plausible models (∆ AIC < 7) explaining sporocarp and ectomycorrhizae species richness.

Explanatory variable	Sporocarp richness	Ectomycorrhizae richness
Forest type	0.27	0.19
Basal area	1.00	0.22
Tree diversity	0.54	0.60
Soil1	0.31	0.15
Soil2	0.23	0.47
Soil3	0.22	0.39
Understorey richness	0.40	0.13

**Table 3:** Indicator values representing associations by forest type, for species with *P* < 0.05.

Forest type	Species	Indicator value	*P*
(a) Sporocarp			
Overmature planted	*Cortinarius flexipes*	0.51	0.008
Old growth	*Hydnum rufescens*	0.41	0.048
(b) Ectomycorrhizae			
Old growth	*Laccaria proxima*	0.55	0.034
