# Supplementary material for: Similar biodiversity of ectomycorrhizal fungi in set-aside plantations and ancient old-growth broadleaved forests
Source: Biol Conserv. 2016 Feb;194:71–9. doi: 10.1016/j.biocon.2015.12.003 (PMC4730558; doi:10.1016/j.biocon.2015.12.003)
Supplement: Supplementary file 1 — Supplementary material. [file mmc1.docx]

# Similar biodiversity of ectomycorrhizal fungi in set-aside plantations and ancient broadleaved forests

Rebecca Spake, Sietse van der Linde, Adrian C. Newton, Laura M. Suz, Martin I. Bidartondo, and C. Patrick Doncaster.

## Supporting Information

Contents

[Appendix A1 – Locations of the seven pairs of ancient and overmature planted forest stands, and sampling design within locations 2](#_Toc435425621)

[Appendix A2 – Details of important environmental variables measured in each plot. 5](#_Toc435425622)

[Appendix A3 – Species accumulation curves at plot- and stand-levels 6](#_Toc435425623)

[Appendix A4 – Lists of species recorded in this study and their ranked abundance distributions. 8](#_Toc435425624)

[Appendix A5 – PCA ordination of soil variables within the 14 ancient and planted forest stands. 15](#_Toc435425625)

[Appendix A6 – Description of PCA axes representing soil chemistry variables in forest stands included in the analysis of EMF richness variation. 16](#_Toc435425626)

[Supplementary information references 19](#_Toc435425627)

### **Appendix A1 – Locations of the seven pairs of ancient and overmature planted forest stands, and sampling design within locations**


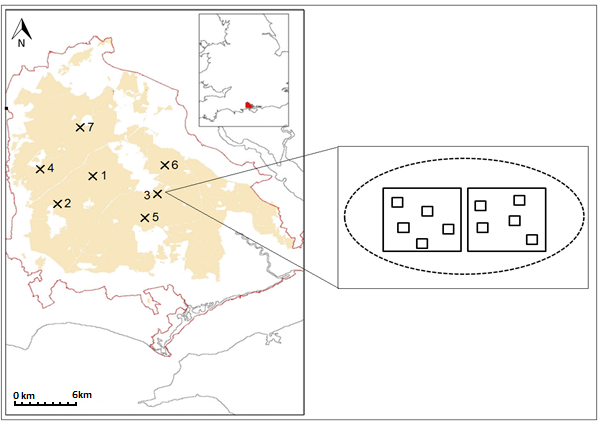


Location

Stand (ancient)

Stand (planted)

Plots

**Figure A1.1.** Simpliﬁed map of New Forest National Park in southern England (inset) showing the distribution of locations (×) across the forested area (coloured) and the arrangement of plots nested in stands nested in each location. Each location consisted of an ancient old-growth and a planted stand, each with five randomly stratified sampling plots, 10×10-m in size.

**Table A1.1.** Details of forest stands used in this study.

| Location | Site name | Stand age / years* | Forest type^§^ | Lat,Long |
| --- | --- | --- | --- | --- |
| 1 | Mark Ash Wood | 10^4^ | A | 50° 52.065', -1° 39.433' |
|  | Bolderwood Grounds | 182 | P | 50° 52.149’, -1°39.387’ |
| 2 | Berry Wood | 10^3^ | A | 50° 50.826', -1° 41.839' |
|  | South Oakley | 160 | P | 50° 50.671', -1° 41.558' |
| 3 | Brinken Wood | 10^3^ | A | 50° 50.796', -1° 36.217' |
|  | Hursthill | 204 | P | 50° 50.488', -1° 35.681' |
| 4 | Redshoot Wood | 10^3^ | A | 50° 52.516', -1° 44.097' |
|  | Great Linford | 164 | P | 50° 52.432', -1° 44.366' |
| 5 | Whitley Wood | 10^3^ | A | 50° 51.099', -1° 34.589' |
|  | New Park Plantation | 192 | P | 50° 50.771', -1° 35.318' |
| 6 | Rushpole | 10^3^ | A | 50° 53.244', -1° 33.748' |
|  | Ironshill | 192 | P | 50° 53.397', -1° 33.149' |
| 7 | Anses | 10^3^ | A | 50° 54.703', -1° 40.517' |
|  | South Bentley | 225 | P | 50° 54.867', -1° 40.206' |

^§^ A: ancient, P: overmature planted

*Planted forest stand ages were established from records of planting in Forestry Commission management plans. Ancient stand ages were estimated from historic maps and consultation with local experts.

NB: the ancient stand from location 2 and the planted stand at location 7 were excluded from the analysis after the PCA of soil chemistry revealed unrepresentative soil conditions within these plots in extreme outliers of EMF richness.

**Details of plantation site preparation procedures in the 1800s in the New Forest National Park**

When ageing a planted forest stand, it is important to consider the methods of plantation establishment. Does the disturbance involved in establishing the plantation constitute a re-setting of forest age? The answer to this depends on the species in question. From an ectomycorrhizal fungal point of view, clear-felling a forest prior to replanting involves the removal of its symbiotic host in a mutualistic relationship, in which the fungus is obligately dependent on the tree host. Clear-felling a site is a major disturbance and therefore constitutes a break in continuity, resetting the age of the forest stand for the ectomycorrhizal fungal (EMF) community. The age of a plantation therefore corresponds to the time since the site stand was replanted if the site was clear-felled.

Details relating to the procedure of inclosure formation (i.e. whether sites were completely clear-felled or not) are patchy and incomplete. There are some forestry records available, including Annual and Triennial Reports of the Comissioners of the Woods, Forests and the Land Revenues of the Crown. The general consensus is that the 19th century plantations made between 1800 and the 1850 (prior to the Deer Removal Act 1851), were clearfelled when formed as a matter of policy (Sanderson 2007). A letter addressed to the Commissioner of Woods, dated 05/12/1816, and titled “Instructions for sundry works to be executed in the New Forest” from Mr Turner, the Deputy Surveyor of the New Forest, sheds some light on the site preparation procedure following the clear-felling of a site. Mr Turner instructs:

*“to dig a trench the ground in beds of about six feet wide each so thrown up as to leave a trench on each side for carrying off the water and leaving a space of about four and a half feet undug between each bed, to plant on the beds that one dug on trenched two rows of trees at the distance of four feet apart in a proportion of about two thirds of strong 4 or 5 year old Oak Plants and one third of Fin Sanch or other fast growing trees best calculated for shelter”.*

There is evidence of the formation of these trenches in several of the plantations in this study, including Bolderwood Grounds, Great Linford Inclosure, New Park Plantation, Ironshill Inclosure and South Oakley Inclosure. The digging of such trenches and associated upturning of the soil would constitute a major disturbance to EMF fungal communities and essentially re-set the age of the forest stand once replanting had occurred.

### Appendix A2 – Details of important environmental variables measured in each plot.

**Table A2.1** Stand-level environmental variables used explanatory variables in models explaining variation in EMF richness. Mean values (and SD) across five 10-m × 10-m plots are given*. See methods for variable descriptions.

| Location | Forest type | Basal area / m^2^ | Understory richness | Canopy closure / % | Tree diversity |
| --- | --- | --- | --- | --- | --- |
| 1 | SN | 102.2 (5.37) | 2.80 (0.45) | 84.4 (4.59) | 0.73 (0.13) |
|  | P | 97.55 (14.11) | 5.00 (2.12) | 74.4 (9.72) | 0.61 (0.35) |
| 2 | SN | 111.37 (3.59) | 4.20 (2.49) | 86.06 (4.80) | 0.43 (0.40) |
|  | P | 92.72 (7.04) | 5.20 (2.95) | 78.16 (9.98) | 0.39 (0.22) |
| 3 | SN | 98.20 (5.23) | 6.00 (2.12) | 72.75 (4.96) | 0.16 (0.21) |
|  | P | 95.01 (6.83) | 3.20 (2.17) | 77.95 (5.27) | 0.02 (0.03) |
| 4 | SN | 114.16 (5.67) | 8.6 (1.14) | 83.77 (4.74) | 0.42 (0.33) |
|  | P | 100.96 (7.22) | 5.40 (3.13) | 80.45 (3.84) | 0.05(0.01) |
| 5 | SN | 95.92 (10.16) | 2.60 (0.89) | 83.57 (6.95) | 0.62 (0.24) |
|  | P | 97.22 (7.14) | 5.00 (1.58) | 83.15 (6.76) | 0.65 (0.24) |
| 6 | SN | 83.80 (10.67) | 4.40 (1.52) | 73.58 (8.53) | 0.21 (0.15) |
|  | P | 99.39 (9.5) | 4.20 (0.45) | 80.90 (5.49) | 0.12 (0.21) |
| 7 | SN | 100.49 (15.72) | 4.8 (2.17) | 80.45 (11.09) | 0.88 (0.22) |
|  | P | 110.21 (9.97) | 2.8 (1.48) | 83.57 (8.17) | 0.61 (0.13) |

*Basal area and tree diversity measurements are from 30-m × 30-m plots (see text).

### Appendix A3 – Species accumulation curves at plot- and stand-levels


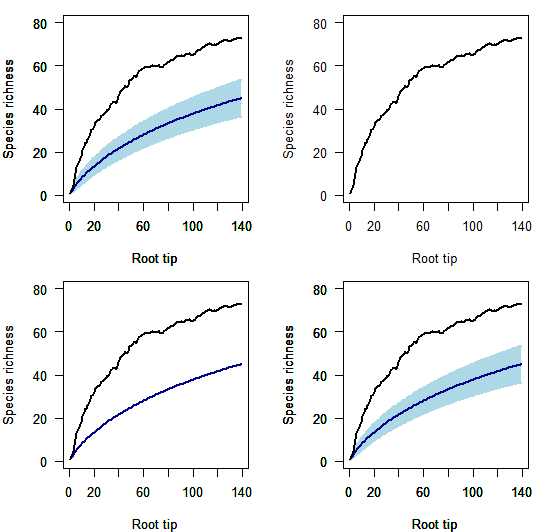


**Figure A3.1.** Species accumulation curves using root-tip sequence data for a planted stand (Location 1). Black = Chao1 non-parametric richness estimator; blue = mean rarefied richness (±CI) where root tips were sampled randomly without replacement using 100 permutations of the data.


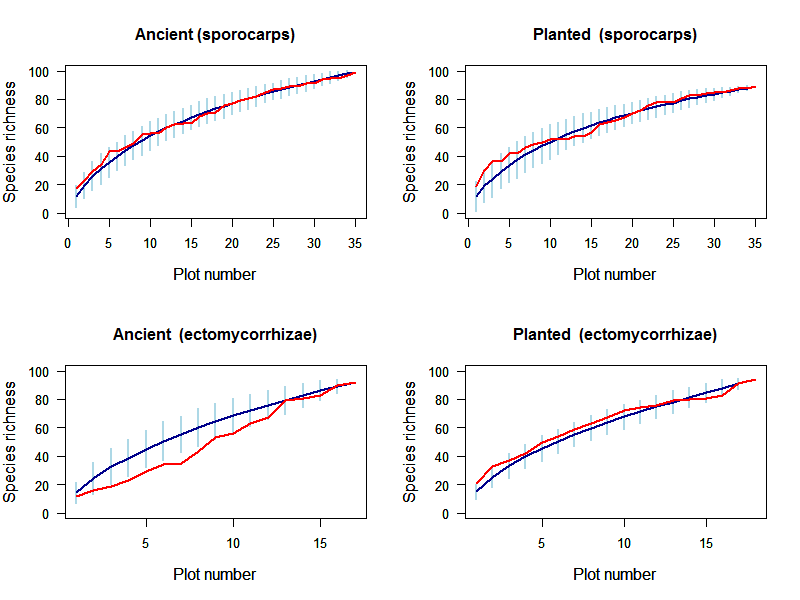


**Figure A3.2.** Species accumulation curves for all planted and ancient forest plots using above-ground (sporocarp) and below-ground (ectomycorrhizae) data. Blue = mean rarefied richness (±CI), with plots sampled randomly without replacement using 100 permutations of the data; red = by order of collection.

### Appendix A4 – Lists of species recorded in this study and their ranked abundance distributions.

**Table A4.1.** The 225 species recorded as ectomycorrhizae and sporocarps in this study, with the number of plots that they were observed in.

|  | Number of plots that the species occurred in | | | |
| --- | --- | --- | --- | --- |
|  | Ancient forest | | Planted forest | |
| Species | Ectomycorrhizae | Sporocarps | Ectomycorrhizae | Sporocarps |
| *Amanita citrina* var*. alba* | 0 | 1 | 0 | 2 |
| *Amanita citrina* | 2 | 9 | 0 | 12 |
| *Amanita constricta* | 0 | 0 | 1 | 0 |
| *Amanita fulva* | 1 | 2 | 1 | 0 |
| *Amanita muscaria* | 0 | 1 | 0 | 0 |
| *Amanita phalloides* | 0 | 1 | 0 | 0 |
| *Amanita rubescens* | 6 | 7 | 3 | 11 |
| *Amanita rubescens* var. *annulosulphurea* | 0 | 1 | 0 | 1 |
| *Amanita vaginata* | 0 | 0 | 0 | 2 |
| *Ascomycota sp.1* | 1 | 0 | 0 | 0 |
| *Ascomycota sp.2* | 1 | 0 | 0 | 0 |
| *Ascomycota sp.3* | 0 | 0 | 1 | 0 |
| *Ascomycota sp.4* | 0 | 0 | 1 | 0 |
| *Boletus aereus* | 0 | 0 | 0 | 1 |
| *Boletus aestivalis* | 1 | 0 | 0 | 0 |
| *Boletus appendiculatus* | 1 | 0 | 1 | 0 |
| *Boletus badius* | 0 | 1 | 0 | 3 |
| *Boletus chrysenteron* | 0 | 1 | 0 | 0 |
| *Boletus edulis* | 0 | 5 | 1 | 1 |
| *Boletus erythropus* | 2 | 3 | 0 | 1 |
| *Boletus pulverentus* | 0 | 0 | 0 | 2 |
| *Cenococcum geophilum* | 8 | 0 | 11 | 0 |
| *Chaetosphaeria sp.1* | 0 | 0 | 1 | 0 |
| *Clavulina cinerea* | 2 | 0 | 2 | 0 |
| *Clavulina sp.1* | 0 | 0 | 1 | 0 |
| *Clavulina sp.2* | 0 | 0 | 1 | 0 |
| *Clavulina sp.3* | 1 | 0 | 0 | 0 |
| *Cortinarius acutus* | 0 | 2 | 0 | 2 |
| *Cortinarius alboviolaceus* | 0 | 3 | 0 | 3 |
| *Cortinarius anomalus* | 0 | 0 | 2 | 0 |
| *Cortinarius anthracinus* | 0 | 1 | 0 | 1 |
| *Cortinarius bolaris* | 0 | 0 | 0 | 1 |
| *Cortinarius brunneus* | 0 | 1 | 0 | 3 |
| *Cortinarius croceus* | 0 | 0 | 0 | 1 |
| *Cortinarius decipiens* | 0 | 9 | 0 | 5 |
| *Cortinarius delibutus* | 0 | 1 | 0 | 0 |
| *Cortinarius diasospermus* | 2 | 0 | 3 | 0 |
| *Cortinarius duracinus* | 0 | 3 | 0 | 1 |
| *Cortinarius duracinus* var*. raphanicus* | 0 | 0 | 1 | 0 |
| *Cortinarius elatior* | 0 | 2 | 0 | 1 |
| *Cortinarius flexipes* | 2 | 1 | 2 | 10 |
| *Cortinarius flexipes* var*. flabellus* | 0 | 1 | 0 | 0 |
| *Cortinarius flexipes* var*. inolens* | 0 | 3 | 0 | 1 |
| *Cortinarius hemitriccus* | 0 | 0 | 0 | 2 |
| *Cortinarius hinnuleus* | 0 | 0 | 1 | 1 |
| *Cortinarius incisus* | 1 | 0 | 0 | 0 |
| *Cortinarius mucifluoides* | 0 | 6 | 0 | 4 |
| *Cortinarius obtusus* | 0 | 0 | 1 | 1 |
| *Cortinarius ocroleucus* | 0 | 1 | 0 | 0 |
| *Cortinarius orellanus* | 0 | 1 | 0 | 0 |
| *Cortinarius paleaceus* | 0 | 1 | 0 | 4 |
| *Cortinarius phaeosmus* | 0 | 0 | 0 | 1 |
| *Cortinarius purpurascens* | 0 | 0 | 0 | 1 |
| *Cortinarius rigens* | 0 | 1 | 0 | 0 |
| *Cortinarius rubellus* | 0 | 0 | 1 | 0 |
| *Cortinarius sanguineus* | 2 | 4 | 0 | 8 |
| *Cortinarius sp.1* | 1 | 0 | 0 | 0 |
| *Cortinarius stillatitius* | 1 | 0 | 0 | 0 |
| *Cortinarius torvus* | 0 | 1 | 0 | 0 |
| *Cortinarius valgus* | 0 | 0 | 1 | 0 |
| *Cortinarius violaceus* | 0 | 1 | 0 | 0 |
| *Cortinarius xanthocephalus* | 0 | 1 | 0 | 0 |
| *Craterellus tubaeformis* | 0 | 2 | 1 | 3 |
| *Dermataceae sp.1* | 0 | 0 | 1 | 0 |
| *Elaphomyces muricatus* | 7 | 0 | 12 | 0 |
| *Entoloma conferendum* | 0 | 0 | 1 | 0 |
| *Exidia sp.1* | 2 | 0 | 2 | 0 |
| *Exidiopsis plumbescens* | 0 | 0 | 1 | 0 |
| *Genea hispidula* | 3 | 0 | 0 | 0 |
| *Gyroporous castaneus* | 0 | 0 | 0 | 1 |
| *Hebeloma birrus* | 0 | 5 | 0 | 5 |
| *Hebeloma crustuliniforme* | 0 | 1 | 0 | 4 |
| *hebeloma edurum* | 0 | 1 | 0 | 0 |
| *Hebeloma hiemale* | 0 | 1 | 0 | 0 |
| *Hebeloma mesophaum* | 0 | 8 | 0 | 7 |
| *Hebeloma pumilum* | 0 | 2 | 0 | 1 |
| *Hebeloma theorbrominum* | 0 | 1 | 0 | 0 |
| *Heliotales sp.1* | 1 | 0 | 0 | 0 |
| *Heliotales sp.2* | 0 | 0 | 1 | 0 |
| *Helotiaceae sp.3* | 0 | 0 | 1 | 0 |
| *Helotiales sp.4* | 0 | 0 | 1 | 0 |
| *Helotiales sp.5* | 1 | 0 | 0 | 0 |
| *Helotiales sp.6* | 0 | 0 | 1 | 0 |
| *Hyaloscyphaceae sp.1* | 1 | 0 | 1 | 0 |
| *Hydnum repandum* | 0 | 3 | 0 | 2 |
| *Hydnum rufescens* | 2 | 7 | 1 | 1 |
| *Hydnum sp.1* | 1 | 0 | 0 | 0 |
| *Hydronata tulasnei* | 4 | 0 | 3 | 0 |
| *Ilyonectria sp.1* | 1 | 0 | 0 | 0 |
| *Inocybe assimilata* | 1 | 0 | 0 | 0 |
| *Inocybe cookei* | 0 | 1 | 0 | 0 |
| *Inocybe fastigiata* | 0 | 1 | 0 | 0 |
| *Inocybe languinosa* | 1 | 0 | 2 | 0 |
| *Inocybe napipes* | 1 | 1 | 1 | 2 |
| *Inocybe pudica* | 0 | 0 | 0 | 1 |
| *Laccaria amethystina* | 9 | 22 | 11 | 29 |
| *Laccaria bicolor* | 0 | 4 | 0 | 10 |
| *Laccaria laccata* | 3 | 14 | 5 | 18 |
| *Laccaria proxima* | 4 | 3 | 1 | 6 |
| *Laccaria tortilis* | 0 | 2 | 0 | 0 |
| *Lactarius blennius* | 1 | 3 | 2 | 1 |
| *Lactarius camphoratus* | 5 | 6 | 7 | 6 |
| *Lactarius chrysorrheus* | 4 | 14 | 9 | 14 |
| *Lactarius decipiens* | 0 | 2 | 0 | 2 |
| *Lactarius evosmus* | 0 | 4 | 0 | 5 |
| *Lactarius flexuosus var roseozonatus* | 0 | 2 | 0 | 0 |
| *Lactarius fulvissiumus* | 0 | 11 | 0 | 10 |
| *Lactarius lapponicus* | 1 | 0 | 0 | 0 |
| *Lactarius quietus* | 16 | 27 | 18 | 25 |
| *Lactarius rufus* | 0 | 2 | 0 | 2 |
| *Lactarius serifluus* | 4 | 1 | 3 | 2 |
| *Lactarius sp.1* | 1 | 0 | 0 | 0 |
| *Lactarius subdulcis* | 5 | 5 | 0 | 4 |
| *Lactarius subdulcis* | 0 | 0 | 0 | 0 |
| *Lactarius subumbonatus* | 0 | 4 | 0 | 2 |
| *Lactarius tabidus* | 14 | 27 | 12 | 28 |
| *Lactarius volemus* | 0 | 2 | 0 | 0 |
| *Leccinum auranticaum* | 0 | 0 | 0 | 1 |
| *Leccinum scabrum* | 0 | 2 | 0 | 0 |
| *Leohumicola minima* | 0 | 0 | 1 | 0 |
| *Leotia lubrica* | 1 | 0 | 0 | 0 |
| *Leotiomycetes sp.1* | 1 | 0 | 0 | 0 |
| *Mortierella gemmifera* | 0 | 0 | 1 | 0 |
| *Mortierella humilis* | 1 | 0 | 0 | 0 |
| *Naucoria bohemica* | 0 | 1 | 0 | 0 |
| *Neobulgaria pura* | 1 | 0 | 0 | 0 |
| *Oidiodendron sp. 1* | 1 | 0 | 4 | 0 |
| *Oidiodendron sp.2* | 0 | 0 | 4 | 0 |
| *Oidiodendron sp.3* | 0 | 0 | 1 | 0 |
| *Pachyphloeus sp.1* | 0 | 0 | 1 | 0 |
| *Paxillus involutus* | 0 | 2 | 0 | 6 |
| *Peziza sp. 1* | 7 | 0 | 4 | 0 |
| *Pezizal sp. 2* | 0 | 0 | 3 | 0 |
| *Phialea sp.2* | 0 | 0 | 1 | 0 |
| *Phialea sp.1* | 1 | 0 | 0 | 0 |
| *Pseudocraterellus sp.1* | 1 | 0 | 1 | 0 |
| *Pseudomentella sp.2* | 1 | 0 | 3 | 0 |
| *Pseudotomentella tristis* | 1 | 0 | 0 | 0 |
| *Russula adusta* | 0 | 1 | 0 | 0 |
| *Russula aeruginea Fr.* | 0 | 1 | 0 | 1 |
| *Russula amoenolens* | 0 | 0 | 4 | 0 |
| *Russula anthracina* | 1 | 1 | 1 | 3 |
| *Russula aquosa* | 0 | 0 | 0 | 1 |
| *Russula atropurpurea* | 5 | 6 | 7 | 2 |
| *Russula betularum* | 0 | 0 | 0 | 1 |
| *Russula brunneoviolacea* | 0 | 1 | 2 | 1 |
| *Russula citrina* | 1 | 0 | 1 | 0 |
| *Russula cyanoxantha* | 1 | 7 | 1 | 7 |
| *Russula cyanoxantha forma pelteraui* | 0 | 1 | 0 | 0 |
| *Russula cyanoxantha* var. *variata* | 0 | 1 | 0 | 1 |
| *Russula densifola* | 4 | 6 | 5 | 4 |
| *Russula farinipes Romell.* | 0 | 5 | 0 | 3 |
| *Russula fellea* | 2 | 6 | 1 | 6 |
| *Russula fragilis* | 10 | 13 | 8 | 9 |
| *Russula graveolens* | 1 | 0 | 2 | 0 |
| *Russula heterophylla* | 1 | 0 | 0 | 0 |
| *Russula langei* | 0 | 1 | 0 | 0 |
| *Russula lutea* | 0 | 1 | 0 | 0 |
| *Russula luteotacta* | 0 | 2 | 0 | 0 |
| *Russula mairei* | 5 | 0 | 5 | 0 |
| *Russula nigricans* | 3 | 8 | 1 | 6 |
| *Russula noblis* | 0 | 9 | 0 | 9 |
| *Russula ochroleuca* | 9 | 16 | 13 | 21 |
| *Russula odorata* | 0 | 0 | 0 | 1 |
| *Russula parazurea* | 0 | 0 | 3 | 0 |
| *Russula pelargonium* | 0 | 2 | 0 | 1 |
| *Russula persicina* | 0 | 0 | 0 | 1 |
| *Russula postiana* | 1 | 0 | 0 | 0 |
| *Russula puellaris* | 0 | 0 | 0 | 1 |
| *Russula purpurobadia* | 0 | 0 | 0 | 1 |
| *Russula raoultii* | 0 | 2 | 0 | 0 |
| *Russula silvestris* | 1 | 6 | 8 | 7 |
| *Russula sp FH CLN16F1SY* | 0 | 0 | 1 | 0 |
| *Russula sp.1* | 0 | 0 | 1 | 0 |
| *Russula sp.2* | 0 | 0 | 1 | 0 |
| *Russula sp.3* | 1 | 0 | 0 | 0 |
| *Russula velenovskyi* | 3 | 0 | 0 | 0 |
| *Russula versicolor* | 0 | 2 | 0 | 1 |
| *Russula vesca* | 4 | 3 | 2 | 1 |
| *Russula violacea* | 0 | 1 | 0 | 0 |
| *Russula violeipes* | 0 | 0 | 1 | 0 |
| *Scleroderma areolatum* | 1 | 1 | 0 | 1 |
| *Scleroderma citrinum* | 0 | 5 | 1 | 6 |
| *Scleroderma verrucosum* | 0 | 1 | 0 | 0 |
| *Scopuloides hydnoides* | 1 | 0 | 0 | 0 |
| *Sebacina sp.1* | 1 | 0 | 1 | 0 |
| *Sebacina sp.2* | 1 | 0 | 0 | 0 |
| *Sebacina sp.3* | 1 | 0 | 0 | 0 |
| *Sebacina sp.4* | 0 | 0 | 1 | 0 |
| *Sistotrema sp.1* | 1 | 0 | 0 | 0 |
| *Telamonia sp.1* | 0 | 0 | 0 | 1 |
| *Thelephoraceae sp.1* | 4 | 0 | 4 | 0 |
| *Thelephoraceae sp.2* | 2 | 0 | 2 | 0 |
| *Thelophoraceae sp 2* | 0 | 0 | 0 | 0 |
| *Thelophoraceae sp.1* | 0 | 0 | 0 | 0 |
| *Tomentella botryoides* | 0 | 0 | 1 | 0 |
| *Tomentella coerulea* | 1 | 0 | 0 | 0 |
| *Tomentella sp.1* | 1 | 0 | 2 | 0 |
| *Tomentella sp.2* | 2 | 0 | 2 | 0 |
| *Tomentella sp.3* | 1 | 0 | 0 | 0 |
| *Tomentella sp.4* | 0 | 0 | 1 | 0 |
| *Tomentella stuposa* | 0 | 0 | 1 | 0 |
| *Tomentella sublilacina* | 5 | 0 | 8 | 0 |
| *Trechisporales sp.1* | 1 | 0 | 0 | 0 |
| *Trechisporales sp.2* | 1 | 0 | 0 | 0 |
| *Trechisporales sp.3* | 0 | 0 | 1 | 0 |
| *Tricholoma columbetta* | 1 | 0 | 0 | 0 |
| *Tricholoma fulva* | 0 | 1 | 0 | 0 |
| *Trichopezizella relicina* | 1 | 0 | 0 | 0 |
| *Tuber puberulum* | 1 | 0 | 1 | 0 |
| *Tylopilus felleus* | 1 | 0 | 0 | 0 |
| *Unidentified sp.1* | 0 | 1 | 0 | 3 |
| *Unidentified sp.2* | 1 | 0 | 1 | 0 |
| *Unidentified sp.3* | 2 | 0 | 1 | 0 |
| *Unidentified sp.4* | 1 | 0 | 0 | 0 |
| *Xerocomellus engeleii* | 0 | 1 | 0 | 0 |
| *Xerocomellus pruinatus* | 6 | 1 | 9 | 0 |
| *Xerocomus badius* | 0 | 0 | 3 | 0 |
| *Xerocomus chrysenteron* | 0 | 0 | 1 | 0 |
| *xerocomus ferrugineus* | 0 | 0 | 0 | 2 |
| *Xerocomus rubellus* | 0 | 0 | 0 | 1 |
| *Xerocomus sp.1* | 0 | 0 | 1 | 0 |
| *Xerocomus submentosus* | 0 | 3 | 0 | 3 |


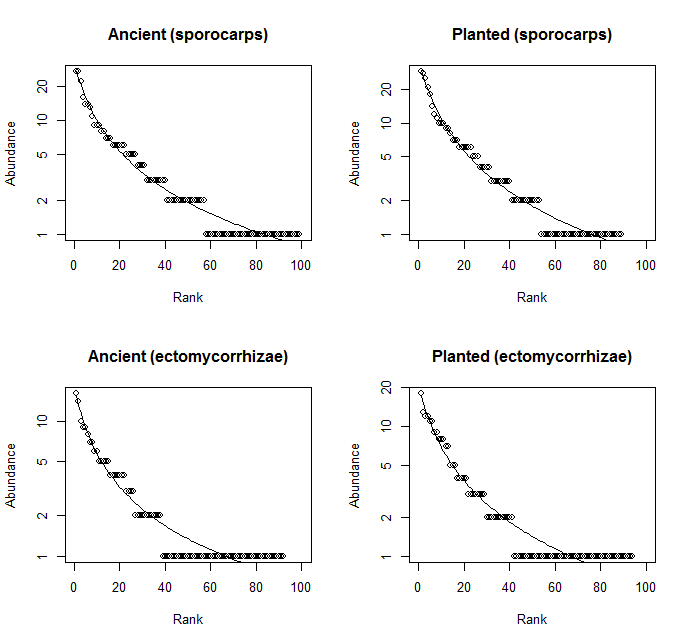
**Figure A4.1**. Ranked abundance distributions of species in ancient and planted forest stands as sporocarps and ectomycorrhizae. Abundance values represent the number of plots that a species occurred in. Rank abundance curves were constructed using the radfit() function of the ‘vegan’ package using the Zipf-Mandelbrot distribution (Oksanen et al. 2013).

### Appendix A5 – PCA ordination of soil variables within the 14 ancient and planted forest stands.


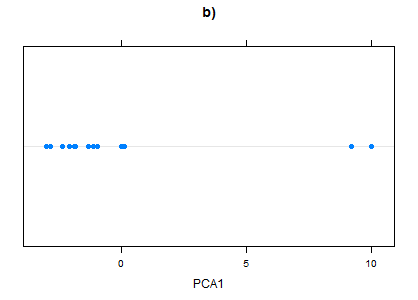

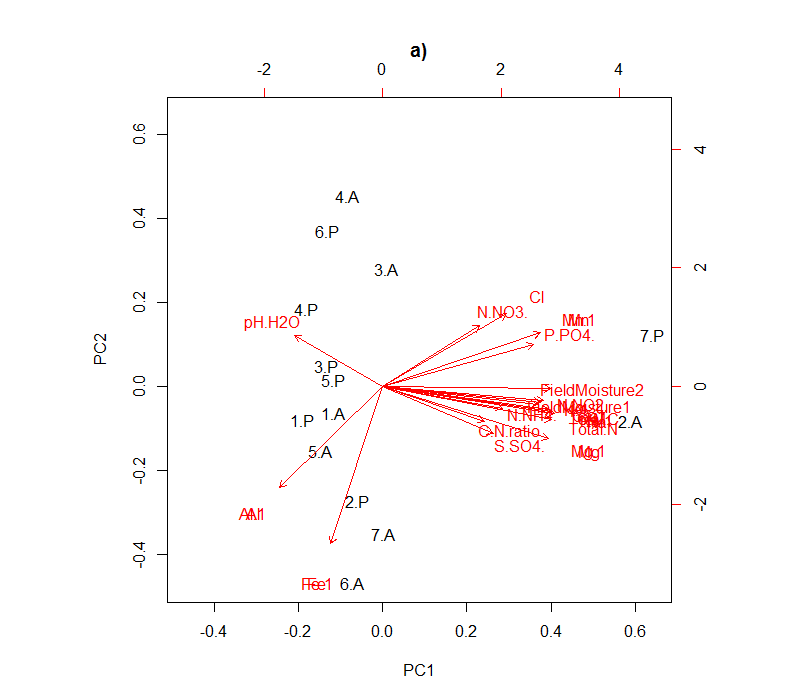


7.P

2.A

**Figure A5.1.** a) PCA ordination of soil variables within the 14 ancient (A) and planted (P) stands located in seven locations used in the study. b) Dotplot of forest stands across the first soil PCA axis. Stand 7.P and 1.A are very different and therefore were excluded from the analysis.

### Appendix A6 – Description of PCA axes representing soil chemistry variables in forest stands included in the analysis of EMF richness variation.


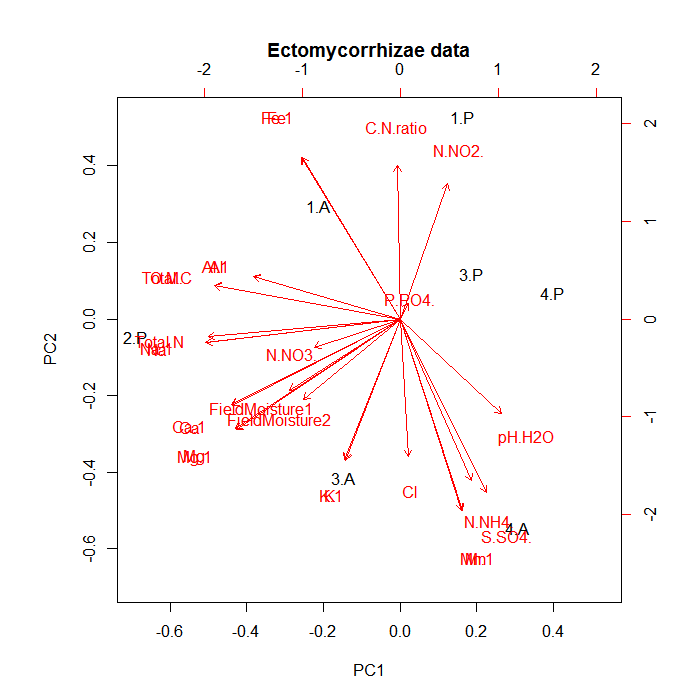

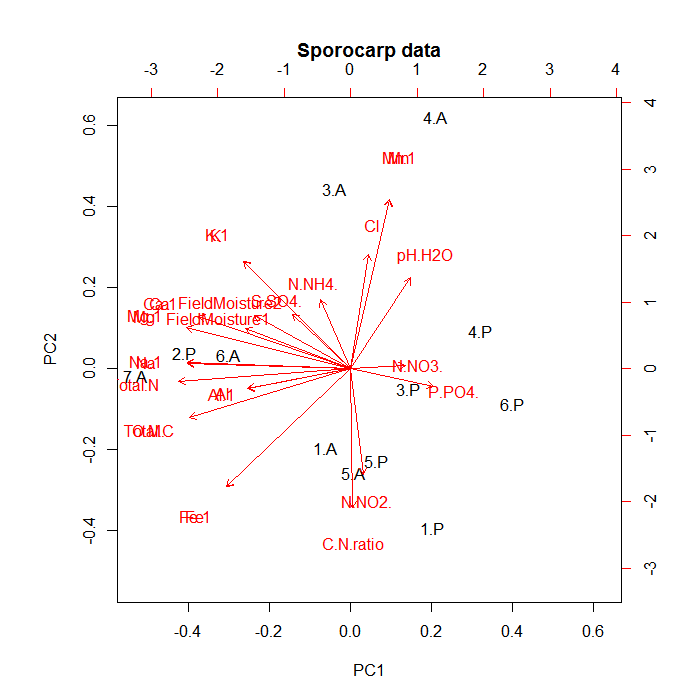


**Figure A6.1**. PCA ordination of soil variables used in analyses of EMF richness variation for the sporocarp and ectomycorrhizae sample datasets.

**Table A6.1.** Soil variable loadings on the three principle components in the forest stands used in analyses of EMF richness variation from sporocarp and ectomycorrhizae surveys. Variance values indicate the percentage of the total variance in soil properties accounted for by each principle component. The cumulative percentage of variance accounted for by the three components summed to 73% and 81% for sporocarp and ectomycorrhizae datasets, respectively.

|  | Sporocarp dataset | |  | Ectomycorrhizae dataset | | |
| --- | --- | --- | --- | --- | --- | --- |
| Soil variable | soil1 | soil2 | soil3 | soil1 | soil2 | soil3 |
| pH | 0.327 | 0.492 | 0.552 | 0.487 | -0.457 | -0.492 |
| Field.Moisture1 | -0.571 | 0.219 | -0.160 | -0.535 | -0.343 | -0.466 |
| FieldMoisture2 | -0.523 | 0.289 | -0.220 | -0.467 | -0.388 | -0.379 |
| Total.N | -0.932 | -0.069 | -0.129 | -0.927 | -0.085 | 0.162 |
| Total.C | -0.871 | -0.265 | -0.197 | -0.897 | 0.162 | 0.084 |
| Organic matter | -0.871 | -0.265 | -0.196 | -0.897 | 0.162 | 0.084 |
| C.N.ratio | 0.013 | -0.755 | -0.105 | -0.013 | 0.741 | -0.189 |
| Cl | 0.097 | 0.622 | -0.334 | 0.038 | -0.664 | -0.020 |
| N.NO3. | 0.296 | 0.017 | -0.417 | -0.416 | -0.136 | -0.060 |
| S.SO4. | -0.317 | 0.296 | 0.785 | 0.416 | -0.837 | -0.238 |
| P.PO4. | 0.449 | -0.098 | -0.244 | 0.042 | 0.076 | 0.930 |
| N.NO2. | 0.072 | -0.575 | 0.689 | 0.229 | 0.653 | -0.593 |
| N.NH4. | -0.162 | 0.373 | 0.651 | 0.344 | -0.782 | 0.402 |
| K | -0.582 | 0.580 | -0.174 | -0.268 | -0.678 | 0.547 |
| Ca | -0.823 | 0.285 | -0.133 | -0.811 | -0.415 | -0.241 |
| Mg | -0.893 | 0.223 | 0.301 | -0.795 | -0.531 | -0.084 |
| Na | -0.887 | 0.028 | -0.379 | -0.938 | -0.114 | 0.301 |
| Al | -0.557 | -0.108 | 0.695 | -0.710 | 0.204 | -0.621 |
| Fe | -0.672 | -0.641 | 0.073 | -0.472 | 0.781 | 0.334 |
| Mn | 0.211 | 0.919 | 0.084 | 0.296 | -0.924 | -0.140 |
| K.1 | -0.579 | 0.583 | -0.173 | -0.264 | -0.681 | 0.548 |
| Ca | -0.823 | 0.284 | -0.133 | -0.812 | -0.415 | -0.241 |
| Mg | -0.892 | 0.226 | 0.301 | -0.792 | -0.535 | -0.089 |
| Na | -0.887 | 0.033 | -0.380 | -0.936 | -0.112 | 0.311 |
| Al | -0.556 | -0.109 | 0.696 | -0.710 | 0.206 | -0.620 |
| Fe | -0.673 | -0.640 | 0.070 | -0.475 | 0.780 | 0.333 |
| Mn | 0.214 | 0.919 | 0.084 | 0.300 | -0.924 | -0.141 |
| Variance / % | 38.3 | 20.4 | 14.4 | 36.4 | 30.2 | 15.1 |

**Table A6.2.** Principle components for soil variables used in analyses

|  | Sporocarp dataset | | | Ectomycorrhizae dataset | | |
| --- | --- | --- | --- | --- | --- | --- |
|  | soil1 | soil2 | soil3 | soil1 | soil2 | soil3 |
| 1.A | -0.692 | -1.602 | -1.006 | -1.768 | 2.228 | -0.320 |
| 1.P | 2.238 | -3.192 | 1.055 | 1.342 | 3.994 | -2.320 |
| 2.P | -4.575 | 0.321 | -1.155 | -5.728 | -0.365 | 0.331 |
| 3.A | -0.449 | 3.609 | -2.018 | -1.235 | -3.140 | -0.737 |
| 3.P | 1.590 | -0.407 | -1.699 | 1.547 | 0.894 | 4.155 |
| 4.A | 2.330 | 5.061 | 1.274 | 2.538 | -4.122 | -1.001 |
| 4.P | 3.581 | 0.751 | 0.877 | 3.305 | 0.512 | -0.107 |
| 5.A | 0.069 | -2.093 | 0.566 | n/a | n/a | n/a |
| 5.P | 0.722 | -1.863 | -0.144 | n/a | n/a | n/a |
| 6.A | -3.350 | 0.284 | 5.068 | n/a | n/a | n/a |
| 6.P | 4.437 | -0.715 | -1.183 | n/a | n/a | n/a |
| 7.A | -5.901 | -0.153 | -1.633 | n/a | n/a | n/a |

### Supplementary information references

Oksanen, J., Blanchet, F. G., Kindt, R., Legendre, P., Michin, P. R., O'hara, R. B., Simpson, P. S., Solymos, P., Stevens, H. H. & Wagner, H. H. 2013. vegan: Community Ecology Package. R package version 2.0-9. <http://CRAN.R-project.org/package=vegan>.

Sanderson, N. A. 2007. New Forest Inclosure habitats, habitat fragmentation and andscape history. Hampshire & Isle of Wight Wildlife Trust, Botley.
